# Remotely-sensed planform morphologies reveal fluvial and tidal nature of meandering channels

**DOI:** 10.1038/s41598-019-56992-w

**Published:** 2020-01-09

**Authors:** Alvise Finotello, Andrea D’Alpaos, Manuel Bogoni, Massimiliano Ghinassi, Stefano Lanzoni

**Affiliations:** 1 Department of Geosciences, University of Padova, via G.Gradenigo, 6, Padova, PD I-35131 Italy; 2 Department ICEA, University of Padova, via Loredan, 20, Padova, PD I-35131 Italy

**Keywords:** Geomorphology, Hydrology

## Abstract

Meandering channels extensively dissect fluvial and tidal landscapes, critically controlling their morphodynamic evolution and sedimentary architecture. In spite of an apparently striking dissimilarity of the governing processes, planform dimensions of tidal and fluvial meanders consistently scale to local channel width, and previous studies were unable to identify quantitative planimetric differences between these landforms. Here we use satellite imagery, measurements of meandering patterns, and different statistical analyses applied to about 10,000 tidal and fluvial meanders worldwide to objectively disclose fingerprints of the different physical processes they are shaped by. We find that fluvial and tidal meanders can be distinguished on the exclusive basis of their remotely-sensed planforms. Moreover, we show that tidal meanders are less morphologically complex and display more spatially homogeneous characteristics compared to fluvial meanders. Based on existing theoretical, numerical, and field studies, we suggest that our empirical observations can be explained by the more regular processes carving tidal meanders, as well as by the higher lithological homogeneity of the substrates they typically cut through. Allowing one to effectively infer processes from landforms, a fundamental inverse problem in geomorphology, our results have relevant implications for the conservation and restoration of tidal environments, as well as from planetary exploration perspectives.

## Introduction

**Figure 1:**
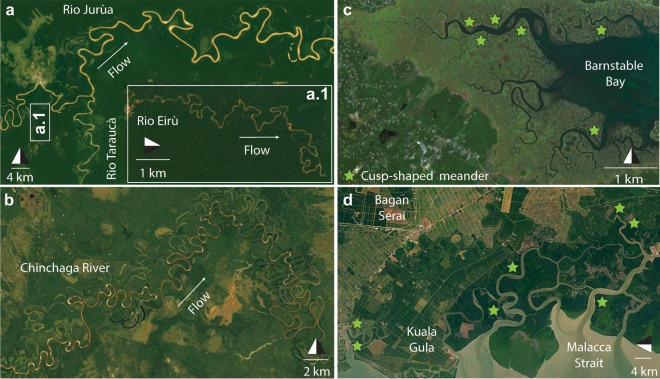
Tidal and Fluvial meanders. (**a**) Confluence of R. Eirú and R. Tarauacá with R. Jurúa, Amazonas (BR). All rivers freely meander for hundreds of kilometers across their alluvial floodplains. A a train of about 50 meanders along R. Eirú is shown in the inset. (Map data: Google, Landsat; 6° 41′S, 69° 44′W). (**b**) Meanders along the Chinchaga River in Alberta (CAN) (Map data: Google, Landsat; 58° 49′N, 118° 21′W). (**c**) Meandering tidal channel networks in Barnstable Bay (MA) displaying densely spaced lateral tributaries, width funneling and peculiar tidal meander morphologies (Map data: Google, TerraMetrics; 41° 43′N, 70° 20′W). (**d**) Tidal meanders along the Malacca Strait coast (Perak, MAL) (Map data: Google, Maxar technologies, CNES/Airbus, TerraMetrics; 4° 53′N, 100° 37′W).

Highly sinuous meandering channels are among the most fascinating morphological patterns existing in nature^1,2^. Their extensive presence typically characterizes both tidal and fluvial landscapes (Fig. 1) and is central in controlling the eco-morphodynamic evolution and sedimentary architecture of the landscapes they cut through^3–7^. Questions concerning analogies and differences among tidal and fluvial meanders (TM and FM hereafter) have long been debated^8–10^, mainly because of the different chief-landforming processes they are formed by. While the basic flow in FM is invariably directed downstream, a periodic flow reversal in response to varying tidal phases controls TM dynamics^3,10^. In addition, maximum flow discharges in fluvial settings are attained when water level is at the top of river banks (i.e., bankfull stage) and generally remain constant along the river course until a major confluence is found. On the contrary, the highest water stages in purely tidal landscapes correspond to low flow conditions (i.e., high slack-water periods), and the maximum along-channel discharges rapidly decline as channels extend landward, leading to a peculiar progressive reduction of channel widths (i.e., channel funneling)^9,11,12^ (Fig. 1).

**Figure 2:**
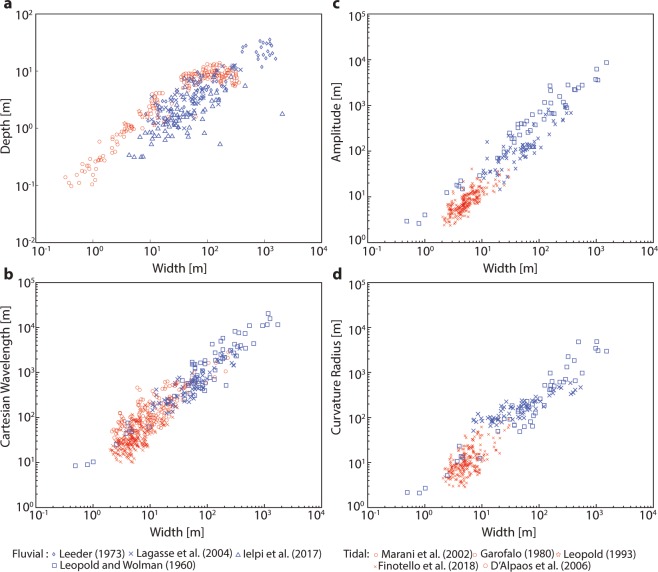
Planform features of tidal and fluvial meanders reported in the literature. (**a**) Width-to-depth ratio of meandering channels. (**b**) Meander width plotted against meander amplitude. (**c**) Meander width plotted against meander cartesian wavelength. (**d**) Meander width plotted against meander curvature radius. Data for fluvial meanders are shown in blue, and were derived from *Leeder*^67^, *Lagasse et al*.^68^, *Ielpi et al*.^69^, and *Leopold and Wolman*^70^. Data for tidal meanders are shown in red, and were derived from *Marani et al*.^9^, *Garofalo*^71^, *D’Alpaos et al*.^72^, *Leopold*^12^, *Finotello et al*.^15^.

**Figure 3:**
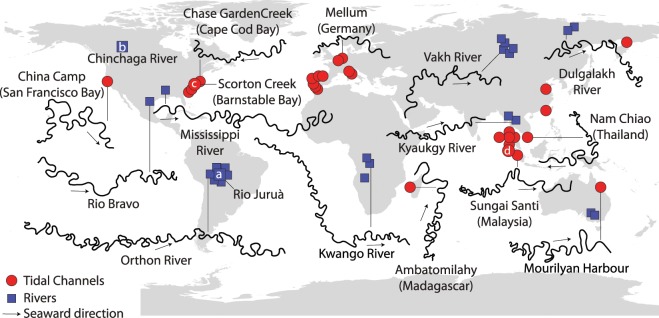
Locations of tidal and fluvial meandering channels considered in this study. Each individual meander in the reported planforms is planimetrically scaled with its average half-width. Letters refer to tidal and fluvial reaches shown in Fig. 1.

In starking contrast to these different chief-landforming processes, however, TM and FM display several remarkable analogies: they are characterized by similar values of sinuosity, as well as ratios between channel width and other relevant features such as mean flow depth, meander amplitude, wavelength and radius of curvature (Fig. 2). The proportionality of relevant morphometric features of TM and FM to channel width, as well as theoretical treatments of the flow field in fluvial and tidal settings^13,14^, also suggests that a proper normalization is ensured by the mean meander width, which allows one to compare meanders of different sizes. Thus, when normalized with local channel width, observed migration rates of TM and FM are shown to be quite similar^15^. In addition, such a scaling substantially mitigates planform morphological differences (e.g., due to channel funneling in tidal environments) and hinders a clear distinction between TM and FM. Looking at the width-normalized planforms of meandering channels shown in Fig. 3, one can hardly disclose their fluvial or tidal nature. Thus far, only sedimentary facies of deposits associated with TM and FM have shown to be different, in spite of apparently similar large scale architecture of TM and FM point bars^16–19^. Differences in sedimentary facies are mainly due to the periodic flow reversal in TM, which produces distinctive sedimentary structures^16,17^. Only in some cases the effect of bidirectional flow significantly impacts the planform shape of individual TM bends. For example, segregated flood and ebb channels, and banks carved in cuspate fashion (Fig. 1c,d), can form as a result of distinct patterns of maximum near-bank velocities attained during ebb and flood^3,16,20^. We therefore wonder whether or not tidal and fluvial meanders consistently display distinct planform features, and to what extent possible differences can be objectively detected. Previous studies were unable to identify suitable, distinctive metrics to quantitatively discriminate between TM and FM planforms^9,10,13^ (Fig. 2), and it still remains unclear whether and how signatures of the different landforming processes operating in tidal and fluvial settings are retained in the corresponding planforms. Developing quantitative frameworks capable of unravelling origins of meandering channels from their planforms might provide remarkable insights into subsurface investigations based on 3D seismic data^21^, and represents a fundamental step to disclose similarities and differences between TM and FM morphodynamics^3,4,9,14^. In addition, it might result crucial for testing different formation theories of extraterrestrial sinuous channels^22,23^, such as those observed in Marsian and Venusian landscapes^24–27^. Here we compare, by means of statistical and spectral tools, the normalized planform features of about 10,000 meander bends observed worldwide, aiming at characterizing differences in footprints of flow fields and morphodynamic processes shaping TM and FM. The detected morphological differences are then interpreted based on a number of available theoretical, numerical, and field studies on meandering channels in order to relate channel morphology to meander dynamics.

## Material

**Figure 4:**
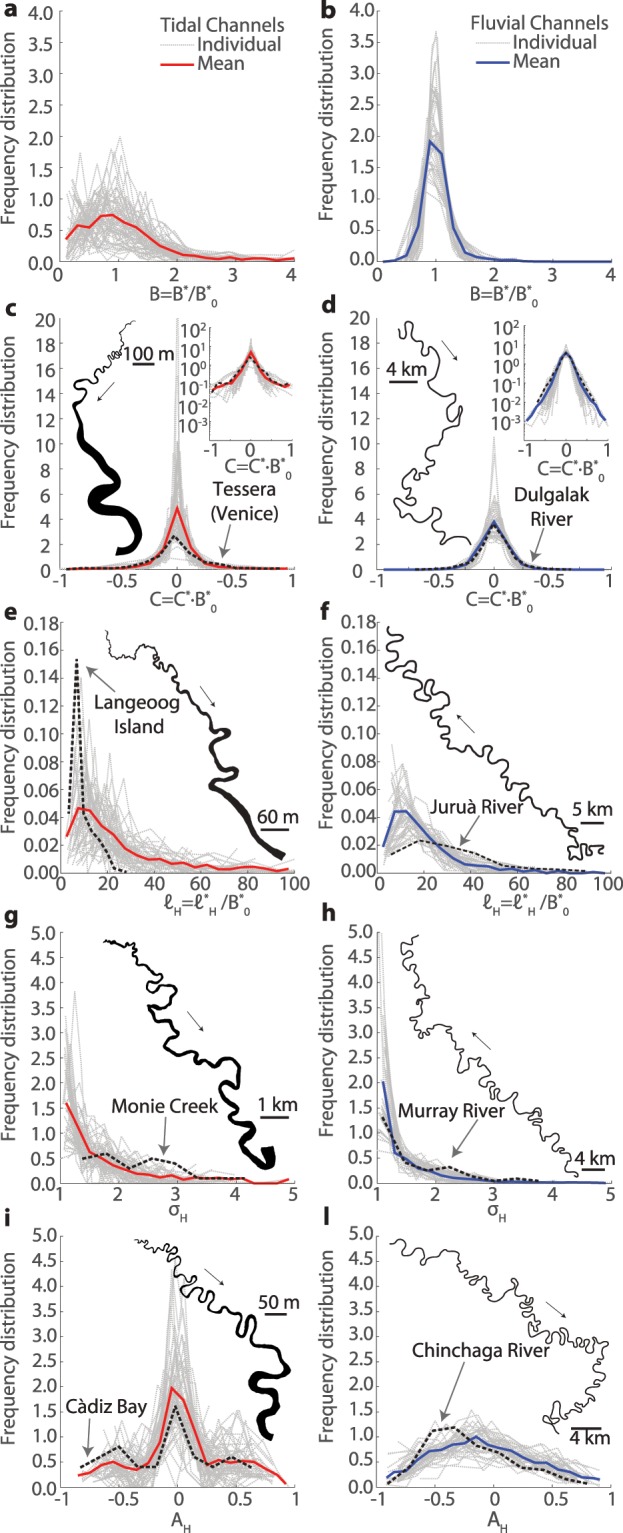
Frequency distributions of some of the normalized morphometric variables considered in this study. (**a,b**) Channel width, (**c,d**) local channel curvature, (**e,f**) half-meander intrinsic length, (**g,h**) half-meander sinuosity, (**i–l**) half-meander asymmetry index. Gray dashed lines represent individual reach data while continuous lines stand for the average frequency distributions. Black dashed lines refer to the channel planform showed in each panel.

A database including 20 different morphometric variables (Table 1) was specifically built by digitizing and analysing satellite images from 38 rivers and 58 tidal channels worldwide (Fig. 3 and see Supplementary Table S1). The analyzed meandering rivers encompass different climate and geological regions, from polar to semiarid, thereby ensuring representativeness for different hydrological regimes, vegetation and land cover, and sediment grainsizes. Similarly, tidal meandering channels included in the dataset are found along coastlines characterized by different tidal regimes, tidal ranges, and vegetation cover, including both vegetated intertidal plains, such as mangrove forests and salt marshes, and intertidal flats devoid of vegetation. In the case of tidal channels, where the length of meander trains is limited by the presence of many bifurcations and confluences, we only considered reaches containing at least 20 consecutive bends. This procedure ensures homogeneity with data from fluvial settings, where the limited number of confluences promotes the formation of much longer meander trains. For example, the Juruá river (Fig. 1a) freely meanders along its 2,414 km long course, whereas the longest tidal channel in Barnstable Bay (Fig. 1c) contains about 60 meander bends and barely covers 5 km in length. To identify individual meander bends and characterize their planform features we employed the method proposed by *Marani et al*.^9^. The procedure is based on the definition of the curve $\Gamma (s)=\{({x}^{\ast }({s}^{\ast }),{y}^{\ast }({s}^{\ast })\}$, where ${s}^{\ast }$ [m] denotes the channel-axis curvilinear coordinate and ${x}^{\ast }({s}^{\ast }),{y}^{\ast }({s}^{\ast })$ represent the coordinates of an arbitrary axis point in a Cartesian reference system. The angle $\theta ({s}^{\ast })$, formed by the tangent to the channel axis and a given arbitrary direction, allows one to compute the local curvature of channel axis (i.e., the inverse of the local radius of curvature), as ${\mathscr{C}}^{\ast }({s}^{\ast })=-\,d\theta ({s}^{\ast })/d{s}^{\ast }$. Fluvial and tidal channel planforms were obtained by digitizing channel banks from satellite images (Fig. 4). Channel axis was derived from a skeletonization procedure and was smoothed by means of a Savitzki-Golay low-pass filter to avoid numerical discontinuities. A uniform discretization grid $\{({x}_{n}^{\ast }({s}_{n}^{\ast }),{y}_{n}^{\ast }({s}_{n}^{\ast })\}$ (${s}_{n}^{\ast }=n\Delta {s}^{\ast },n=\mathrm{0,1,}\,\ldots ,N$) was obtained using standard cubic spline-fit polylines. The angle $\theta ({s}^{\ast })$ and the curvature ${\mathscr{C}}^{\ast }({s}^{\ast })$ were computed numerically in correspondence of the $\Delta {s}^{\ast }$ equally-spaced *N* grid points^28^. Half (full) meander bends were defined as the channel portion between two (three) consecutive inflexion points, i.e. points where ${\mathscr{C}}^{\ast }\mathrm{=0}$. In order to characterize meander planforms, three fundamental metrics were introduced, with respect to either half- or full-meander bends: meander intrinsic wavelength (${\ell }^{\ast }$), meander sinuosity (*σ*), and meander asymmetry index (${\mathscr{A}}$)^9,28,29^. The intrinsic wavelength (${\ell }^{\ast }$) represents the length of a given meander computed along the curvilinear coordinate ${s}^{\ast }$, while sinuosity is defined as the ratio between ${\ell }^{\ast }$ and the meander cartesian wavelength ${L}_{xy}^{\ast }$ (the planar distance between meander end-points). The asymmetry index is computed as ${\mathscr{A}}=({\ell }_{u}^{\ast }-{\ell }_{d}^{\ast })/{\ell }^{\ast }$, where ${\ell }_{u}^{\ast }$ and ${\ell }_{d}^{\ast }$ are the distances between the meander apex and its upstream and downstream end-points, respectively. It is worthwhile noting that all the relevant dimensional variables were normalized with the mean meander half-width ${B}_{0}^{\ast }$ [m] (${\mathscr{C}}={\mathscr{C}}^{\ast }{B}_{0}^{\ast };s={s}^{\ast }/{B}_{0}^{\ast };\ell ={\ell }^{\ast }/{B}_{0}^{\ast };{L}_{xy}={L}_{xy}^{\ast }/{B}_{0}^{\ast }$) to directly compare bends of different sizes.

**Table 1:** Suite of morphometric variables used to objectively characterize planform meandering patterns.

Morphometric variable	Symbol	Description
Sinuosity	*σ*_*av*_	mean meander sinuosity
	*σ*_*va*_	variance of meander sinuosity
	*σ*_*std*_	standard deviation of meander sinuosity
	*σ*_*sk*_	skewness of meander sinuosity
	*σ*_*kr*_	kurtosis of meanders sinuosity
Intrinsic wavelength	${\ell }_{av}$	mean meander intrinsic length
	${\ell }_{va}$	variance of meander intrinsic length
	${\ell }_{std}$	standard deviation of meander intrinsic length
	${\ell }_{sk}$	skewness of meander intrinsic length
	${\ell }_{kr}$	kurtosis of meander intrinsic length
Curvature	${\mathscr{C}}_{av}$	mean local channel curvature
	${\mathscr{C}}_{va}$	variance of local channel curvature
	${\mathscr{C}}_{std}$	standard deviation of local channel curvature
	${\mathscr{C}}_{sk}$	skewness of local channel curvature
	${\mathscr{C}}_{kr}$	kurtosis of local channel curvature
Asymmetry index	${\mathscr{A}}_{av}$	mean meander asymmetry index
	${\mathscr{A}}_{va}$	variance of meander asymmetry index
	${\mathscr{A}}_{std}$	standard deviation of meander asymmetry index
	${\mathscr{A}}_{sk}$	skewness of meander asymmetry index
	${\mathscr{A}}_{kr}$	kurtosis of meander asymmetry index

## Results

### Planform characteristics of tidal and fluvial meanders

Focusing on individual morphometric variables, we firstly find that TM display a higher variability of normalized widths ($B={B}^{\ast }/{B}_{0}^{\ast }$) relatively to FM (Fig. 4a,b), due to their characteristic width funneling. The frequency distribution of local channel curvature (${\mathscr{C}}={\mathscr{C}}^{\ast }{B}_{0}^{\ast }$) (Fig. 4c,d) appears to be less peaked in FM, for which a greater variance of ${\mathscr{C}}$ (1.40 × 10^−2^) is observed relatively to the tidal configurations (0.26 × 10^−2^). These results point at higher morphological complexity of FM planforms, where the curvature widely fluctuates around its mean value. Conversely, TM typically display either extremely high or, more commonly, low curvature values and intermediate curvature reaches are less likely to be observed. Indeed, heavier tails characterize the ${\mathscr{C}}$ distribution of TM (see inset of Fig. 4c), which displays a kurtosis (4.35) higher than FM (0.98). This observation is consistent with empirical evidence showing that tidal channels often follow a straight-meandering-straight pattern, with low curvature observed in both the most landward (where tidal channels are generally younger) and seaward (where wide, poorly sinuous meanders are found) portions of the channel^9^ (Fig. 4c). On the contrary, long and poorly sinuous reaches are likely less frequent in single-thread meandering rivers, where complex, asymmetrical compound meanders are widespread^30^ (Fig. 4d).

Analyses of individual meander morphometric features, carried out by means of a two-sided Kolmogorov-Smirnov (KS) test (*α* = 0.05), demonstrate that the intrinsic length of half meanders (${\ell }_{H}$, Fig. 2e,f), as well as the full-meander intrinsic length (${\ell }_{F}$) and sinuosity (*σ*_*F*_), come from the same distribution for both TM and FM (Table 2). On the contrary, a statistically significant difference between TM and FM is observed for local channel curvature (${\mathscr{C}}$, Fig. 4c,d), half-meander sinuosity (*σ*_*H*_, Fig. 4g,h), full- and half-meander asymmetry index (${\mathscr{A}}_{H}$, Fig. 4i,h) (Table 2). The results concerning ${\mathscr{A}}_{H}$ are of particular interest. The landward (${\mathscr{A}}_{H}\,\mathrm{ < }\,0$) or seaward (${\mathscr{A}}_{H}\,\mathrm{ > }\,0$) skewing of tidal meanders was suggested to reflect ebb- or flood- dominance, respectively, due to the observation that FM, shaped by downstream directed flows, are usually upstream skewed^10,16^ (${\mathscr{A}}_{H}\,\mathrm{ < }\,0$). However, fluvial meander theory suggests that skewing of FM depends on their sub- or super-resonant morphodynamic regime^14^. Sub-resonant conditions, attained for values of the channel width-to-depth ratio (*β*) smaller than the resonant value (*β* < *β*_*R*_), lead to upstream skewed, downstream migrating meanders, while super-resonant conditions (*β* > *β*_*R*_) promote the development of downstream skewed and upstream migrating meanders^14^. Figure 4 shows the presence of both upstream (${\mathscr{A}}_{H}\,\mathrm{ < }\,0$) and downstream (${\mathscr{A}}_{H}\,\mathrm{ > }\,0$) skewed FM, although the former are more frequent in the analysed dataset. We therefore point out that the dominant tidal regime of TM cannot be inferred on the basis of their planform skewing. Indeed, the mean frequency distribution of ${\mathscr{A}}_{H}$ shows the overall absence of a preferred skewing direction of TM (Fig. 4i).

**Table 2:** Results of Kolmogorv-Smirnov test on the selected morphometric variables. Significance level (*α* = 0.05) is constant.

Variable	Null Hyp.	Alt. Hyp.	Rejected	p-value
	*H*_0_	*H*_1_	Null Hyp.	
${\ell }_{{H}_{av}}$	cdf_tidal_ = cdf_fluvial_	cdf_tidal_ ≠ cdf_fluvial_	No	1.70*e*^−1^
${\mathscr{A}}_{{H}_{av}}$	cdf_tidal_ = cdf_fluvial_	cdf_tidal_ ≠ cdf_fluvial_	Yes	8.60*e*^−15^
	cdf_tidal_ = cdf_fluvial_	cdf_tidal_ > cdf_fluvial_	No	9.80*e*^−1^
	cdf_tidal_ = cdf_fluvial_	cdf_tidal_ < cdf_fluvial_	Yes	4.30*e*^−15^
${\sigma }_{{H}_{av}}$	cdf_tidal_ = cdf_fluvial_	cdf_tidal_ ≠ cdf_fluvial_	Yes	1.30*e*^−2^
	cdf_tidal_ = cdf_fluvial_	cdf_tidal_ > cdf_fluvial_	No	1.00*e*^+0^
	cdf_tidal_ = cdf_fluvial_	cdf_tidal_ < cdf_fluvial_	Yes	6.43*e*^−4^
${\ell }_{{F}_{av}}$	cdf_tidal_ = cdf_fluvial_	cdf_tidal_ ≠ cdf_fluvial_	No	1.84*e*^−1^
${\mathscr{A}}_{{F}_{av}}$	cdf_tidal_ = cdf_fluvial_	cdf_tidal_ ≠ cdf_fluvial_	Yes	4.95*e*^−2^
	cdf_tidal_ = cdf_fluvial_	cdf_tidal_ > cdf_fluvial_	Yes	2.47*e*^−2^
${\sigma }_{{F}_{av}}$	cdf_tidal_ = cdf_fluvial_	cdf_tidal_ ≠ cdf_fluvial_	No	3.72*e*^−1^
${\mathscr{C}}_{av}$	cdf_tidal_ = cdf_fluvial_	cdf_tidal_ ≠ cdf_fluvial_	Yes	2.13*e*^−2^
	cdf_tidal_ = cdf_fluvial_	cdf_tidal_ > cdf_fluvial_	Yes	2.13*e*^−2^

### Meander dynamics in tidal and fluvial environments

**Figure 5:**
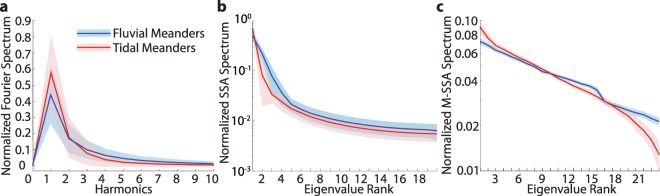
Results of spectral analysis methods. (**a**) Fourier spectra of full-meander curvature ${\mathscr{C}}_{F}$. (**b**) SSA eigenvalue spectra obtained analysing full-meander curvature with a moving window of size *M* = 20. (**c**) M-SSA eigenvalue spectra obtained considering *M* = 8 consecutive half meanders. The units of abscissa in (**b,c**) are SSA and M-SSA component numbers (eigenvalue rank), respectively, while the ordinate shows the variance contributed by each SSA and M-SSA component. Continuous lines represent average values. Displayed intervals correspond to one standard deviation.

In order to characterize the underlying dynamics of meander planforms, we Fourier transformed the curvature signal of all the considered full meanders (${\mathscr{C}}_{F}$), and derived the corresponding power spectra (Fig. 5a and see Methods). In general, most of the spectral power is contained within the first three/four harmonics, while higher order harmonics exhibit a progressive decrease of spectral power density. A physical interpretation of these results may be provided by considering the Kinoshita’s curve^31^, that describes the spatial distribution of channel axis curvature typical of fluvial meandering patterns:

1 $${\mathscr{C}}(s)={\mathscr{C}}_{0}[\cos (\lambda \,s)-{c}_{F}\,\cos \,\mathrm{(3}\lambda s)-{c}_{S}\,\sin \,\mathrm{(3}\lambda s)]$$

where $\lambda \mathrm{=2}\pi /{\ell }_{F}$ is the meander wavenumber, while *c*_*F*_ and *c*_*S*_ represent the fattening and skewing coefficients, respectively. The lack of even harmonics, particularly of the second one, in Eq. (1) was justified by the cubic geometric nonlinearity of the integro-differential equation describing the planimetric-evolution of meandering rivers^14^. Nevertheless, our results highlight the presence of non negligible second harmonics in both TM and FM spectra, with almost identical power density (Fig. 5a). This proves that, unlike previously suggested^9^, TM and FM cannot be distinguished based on the possible presence of even harmonics. In general, TM display higher power density within the first harmonic, followed by a more rapid decay of spectral energy within higher modes compared to FM. Our results indicate a lower complexity of TM planforms, whereas FM are likely to exhibit more complex patterns owing to the marked convolutive (nonlinear) interactions among spectral modes. We applied the Singular Spectrum Analysis^32^ (SSA, see Methods) to full-meander curvature series to further substantiate such hypothesis^29^. The resulting eigenvalue spectra (Fig. 5b) provide information on the separation between the meaningful part of the signal and the noisy background, the latter being mainly confined in the spectrum tail (i.e., components from the 5^th^ to 20^th^). The significance of each spectrum component can be quantitatively estimated by comparing the variance contributed by each SSA eigenvalue. Even though an exponential decay is observed for both fluvial and tidal spectra, the former exhibit a more distinct slope break that usually corresponds to signal to noise (S/N) separation^32^. In addition, the relative importance of each SSA spectrum component, with the sole exception of the first one, is higher in the fluvial than in the tidal case, with the most marked difference observed for the second and third components (Fig. 5b). The slower decay of the SSA spectrum of FM points at a higher morphological complexity relatively to TM, due to the increased weight of high-order spectrum components. The same line of reasoning is supported by the results obtained from the multivariate extension of SSA (M-SSA, see Methods). We employed M-SSA to investigate the planform homogeneity among series of *M*-adjacent half-meanders, with *M* equal to 2, 5, and 8. Regardless of *M*-size, the M-SSA spectra obtained for TM (Fig. 5c and Supplementary Fig. S1) display a faster decay than FM. Increasing the window length *M*, the eigenvalue spectra of FM show, on average, a progressively clearer break, standing for S/N separation, that is conversely absent in TM. Hence, FM belonging to a series of consecutive bends are more likely to display morphological features different from each other, whereas in tidal realms the characteristics of a given individual meander are more similar to those of the adjoining bends.

### Quantifying differences between distinct meander species

**Figure 6:**
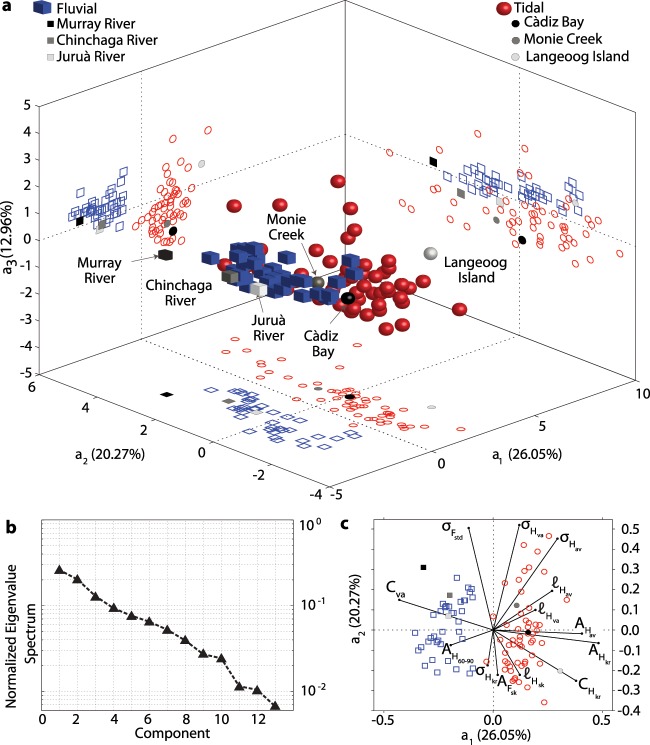
Results of Principal Component Analysis (PCA). (**a**) 3D-reduced space score plot resulting from PCA. The percent of variance explained by each PC is reported along the corresponding PC axis. (**b**) Eigenvalue spectrum of the correlation-matrix. (**c**) Biplot of PC loadings and scores. In order to fit in the loading space, PC scores are divided by the maximum absolute value of all scores and multiplied by the length of the loading vectors.

Despite providing us with a statistical characterization of TM and FM, the analyses of both frequency distributions of individual morphometric variables and curvature spectra do not ensure a clear and univocal quantitative discrimination between the two types of bends. Hence, we further investigated differences and similarities between TM and FM planforms through Principal Component Analysis (PCA), a widely adopted approach in the literature^28,29,33^ (see Methods). The goal of the PCA is to transform the data into a lower-dimensional space while retaining as much as possible of the variation present in the original dataset. Since some of the considered morphometric variables are not strictly independent, a subset of them needs to be considered for characterizing the complexity of TM and FM planforms while avoiding redundant information. Specifically, we considered a subset of 13 variables including: mean, variance, and skewness of half-meander intrinsic wavelength (${\ell }_{{H}_{av}},{\ell }_{{H}_{va}},{\ell }_{{H}_{sk}}$); mean, kurtosis, and 60–90 length percentile of half-meander asymmetry index (${\mathscr{A}}_{{H}_{av}},{\mathscr{A}}_{{H}_{kr}},{\mathscr{A}}_{{H}_{60-90}}$); skewness of full-meander asymmetry index (${\mathscr{A}}_{{F}_{sk}}$); mean, variance, and kurtosis of half-meander sinuosity (${\sigma }_{{H}_{av}},{\sigma }_{{H}_{va}}{\sigma }_{{H}_{kr}}$); standard deviation of full-meander sinuosity (${\sigma }_{{F}_{std}}$); kurtosis of half-meander curvature (${\mathscr{C}}_{{H}_{kr}}$); variance of the local channel curvature (${\mathscr{C}}_{va}$). Results show that this ensemble of morphological parameters can quantitatively discriminate among different patterns of TM and FM. Two well clusterized groups can be observed from the three-dimensional scatterplot of principal component scores, which are the projections of the original data into the space of the principal components (PCs) (Fig. 6a). The first three PCs (*a*_1_, *a*_2_, *a*_3_), which are given by the eigenvectors corresponding to the first three largest eigenvalues of the correlation matrix of data (see Methods), account for about 60% of the observed total variance. The relative importance of higher-order PCs decays exponentially as suggested by the normalized PC eigenvalue spectrum (Fig. 6b). The PCA yields a clear separation between TM and FM, particularly in the {*a*_1_; *a*_2_} and {*a*_1_; *a*_3_} planes. Points corresponding to FM are localized in the negative *a*_1_ half-plane, and exhibit an opposite behaviour relatively to the TM cluster (Fig. 6a). Furthermore, the analysis of PC loadings (i.e., the correlation coefficients defining PCs; Fig. 6c) indicates that FM are characterized by stronger curvature variance (${\mathscr{C}}_{va}$) and lower values of half-meander asymmetry index (${\mathscr{A}}_{{H}_{av}}$). The latter observation confirms the occurrence of overall more complex, strongly skewed meanders in riverine frameworks. Conversely, the higher kurtosis values displayed by both tidal half-meander curvature (${\mathscr{C}}_{{H}_{kr}}$) and asymmetry index (${\mathscr{A}}_{{H}_{kr}}$) (Fig. 6c) suggests that strongly curved and asymmetric bends only occasionally occur in tidal environments^34^, the great majority of TM displaying weakly curved, symmetric planforms on the average.

Besides allowing for a quantitative and visually clear differentiation between TM and FM, the PCA corroborates results of both univariate and multivariate statistical analyses, carried out on both individual TM and FM morphological variables and ensembles thereof. In fact, the PCA clearly shows that a complete set of suitable statical variables is needed to definitively disclose differences between the two species of bends, suggesting that such differences depend upon a complex array of morphodynamic processes characterizing tidal and fluvial landscapes.

## Discussions

Our results prove that TM planforms display less complex morphologies and more regular dynamics than FM, at both the single-meander and the reach length scale. We suggest that the observed diversity between TM and FM is largely due to differences in external forcings and intrinsic characteristics of the environments they wander through, as well as interactions thereof. In what follows, we argument this hypothesis based on review and physical interpretations of the ample literature on meandering channels, encompassing theoretical, numerical, and field studies, especially within the riverine framework^1–10,13–22^.

First, the lower morphological complexity exhibited by TM is likely related to the higher temporal regularity of the chief landforming processes they are carved by. The periodic reversal of tidal currents, together with the well defined range within which tidal discharges can vary, shape TM in a more symmetric fashion relatively to FM, where flow discharges are highly variable and bankfull conditions are attained only during major flood events with an approximately annual frequency^35^. Hence, the combined action of periodic ebb and flood flows tends to shape symmetric meander planforms^13^, providing explanation for the overall absence of a preferred skewing of TM. Deviations from this trend can however arise from local asymmetries of tidal flows^36^, which also support the heavy tails displayed by the distribution of half-meander asymmetry index (${\mathscr{A}}_{H}$, Fig. 4i).

Second, the much higher frequency of large curvature values characterizing TM points at the presence of sharp bends interspersed in mild curvature reaches (insets of Fig. 4c,d), and correlates well with the higher threshold of bank erosion displayed by TM compared to FM. Indeed, intertidal platforms are typically characterized by a more massive presence of cohesive sediments than alluvial plains^3,17,37,38^, where meandering channels commonly rework older non-cohesive channel-belt deposits^4,39^, especially in low accomodation settings^40^. In addition to sediment cohesion, higher erosion-resistance of tidal channel banks is ensured by both sediment autocompaction and stabilization provided by halophytic vegetation^34^, especially in mangrove forests^41,42^. Higher critical threshold for bank erosion in tidal realms implies that active channel migration occurs only in the sharpest portions of a given bend, in contrast with alluvial rivers where bank erosion holds a quasi-linear proportionality to channel curvature, thus typically affecting the entire meander length^34,43^.

Third, mechanical properties of channel banks are intrinsically more variable in alluvial than in intertidal plains, due to both biotic and abiotic factors^44,45^. In tidal environments, most of the sedimentary products are related to tidal inundation and organic production^10,46^, and a more homogeneous soil texture is expected, owing to the continual sediment redistribution operated by tidal currents and to the quite homogeneous grainsize of the transported sediments^47^. In contrast, fluvial floodplains display a higher variability in terms of riparian flora and fauna^48–50^, pedogenic processes^51–53^, and both floodplain lithology and stratigraphy^5,19,54^. Moreover, self-formed lithological heterogeneities (i.e., generated by meander migration itself) of the plains hosting meandering channels are more pronounced in fluvial than in tidal landscapes, due to the greater capacity that meandering rivers have to autogenically modify their on floodplains (e.g., crevasse splays and oxbow lakes)^5,55,56^. These insights are consistent with empirical observation showing that the amount of sediment reworked by channel migration in alluvial plains carved by highly dynamic, single-thread meandering rivers is two to three times larger than in marsh plains^5,10,16^. Both inherited and self-formed heterogeneities crucially modify lateral migration processes^4,43,57^ and, ultimately, the planform shape of meander bends^55^. Recent numerical studies on FM morphodynamics^29^ proved that differences in both SSA and M-SSA spectra, similar to those we observed in this study (Fig. 5b,c), can be attributed to different floodplain heterogeneities. In particular, the presence of widespread scattered self-formed heterogeneities, such as crevasse-splay and point-bar deposits, as well as erosion-resistant clay plugs originated by the infilling of abandoned channels and oxbow lakes^6^, was found to produce more complex meander planforms. In contrast, less complex meander planforms were generated in more homogeneous floodplains similar those found in tidal realms^29^. Hence, the higher morphological complexity displayed by FM is justified by the high heterogeneities of alluvial plains, that triggers the development of complex and compound meander bends^5,58,59^, which are in contrast typically lacking in tidal environments.

Finally, the characteristic high channel density of intertidal plains^60^ further limits TM dynamics, preventing them from freely migrating and fully developing before capturing adjoining channel^61,62^. Channel piracies prevent the late-stage growth of TM, hampering the development of complex and highly sinuous bends and further limiting the lateral reworking of intertidal plain deposits by meander migration. The lower sinuousities and less complex planforms displayed by TM (Figs. 4–6) support the existance of such a mechanism, which also operates in multi-thread sinuous anastomosed rivers, where the large number of reaches increases floodplain drainage density, allowing for frequent channel piracies and limiting meander sinuosity^63,64^.

## Conclusions

A set of statistical analyses was applied to quantitatively differentiate the remotely-sensed planforms of about 10’000 fluvial and tidal meanders worldwide. The use of different uni- and multi-variate statistics allowed us to show that tidal meanders are less morphologically complex and exhibit more regular planform dynamics than their fluvial counterparts. Although it still remains unclear whether or not tidal meanders arise from the same morphodynamic mechanisms observed in rivers^13,14^, our results clearly demonstrate that their later evolution is different and leads to distinct planform morphologies. A comparison of our empirical results against existing observations and data from the literature, supports the idea that the observed planform differences retain signatures of the different chief-landforming processes characterizing fluvial and tidal landscapes. This is due to fundamental differences between tidal and fluvial hydrodynamics, as well as to the peculiar mechanisms of evolution that typifies intertidal areas, where lateral channel reworking is limited and periodic floodings of intertidal areas enhance inorganic deposition, creating more homogeneous substrates relatively to alluvial plains. These findings recapitulate and unify results of previous studies on meander morphodynamics carried out separately in the fluvial and tidal frameworks, and also provide new insights on the morphodynamics of tidal meanders, which escaped the close scrutiny thus far devoted to fluvial meanders. Although morphologies of specific individual bends may differ from the above depicted trends, possibly due to site-specific conditions (e.g., bank erodibility, nonlinear flow dynamics, bioturbations), we deem that the broad range of climate and geological contexts analysed here warrants a proper statistical treatment, and reinforces the significance of the interpretation we propose.

We also demonstrate that adequate metrics exist, capable of characterizing the morphological differences between TM and FM, and allowing one to distinguish them on the exclusive basis of their remotely-sensed planforms. On the one hand, this is a notable achievement per se, representing a critical step to bridge the gap in current knowledge between tidal and fluvial meander morphodynamics^9,23^, with direct implications for restoration purposes^65^. On the other hand, it also provides valuable tools for better understanding the structure of other meandering processes across Earth and planetary sciences^66^, such as those observed in turbidity currents, lava flows, supraglacial streams, and extraterrestrial flows^24,25^. Inferring processes from remotely-sensed landforms represents a challenging and timely research in view of the ever-increasing amount and resolution of Earth and planetary imageries, with critical implications for improving our understanding of geomorphological processes, especially on extraterrestrial planetary surfaces^22,26,27^.

## Methods

### Fourier analysis

The Discrete Fourier Transform (DFT) was used to transform the full-meander curvature signal from the physical domain ${\mathscr{C}}_{F}({s}_{n{\prime} })$ (${s}_{n{\prime} }=n{\prime} /\Delta s,n{\prime} \,\mathrm{=}\,\mathrm{0,}\,\mathrm{1,}\ldots ,N{\prime}$) into the wavenumber domain, yielding a set of *N*′ harmonic components defined as:

2 $${\hat{\mathscr{C}}}_{k}=\mathop{\sum }\limits_{n{\prime} \mathrm{=0}}^{N{\prime} -1}{\mathscr{C}}_{F}({s}_{n{\prime} }){e}^{-i\frac{2\pi n{\prime} }{N{\prime} }k}$$

where *k* was used to sort the harmonics in the wavenumber domain. Since the DFT treats the signal as periodic of period ${\ell }_{F}$, the evaluation of ${\hat{\mathscr{C}}}_{k}$ was carried out for the fundamental wavenumber $\Delta k=(N{\prime} \Delta s{)}^{-1}={\ell }_{F}^{-1}$ and its harmonics ($k=\mathrm{1,}\ldots ,N{\prime} -1$). It is finally worth mentioning that every full meander in our dataset was considered as a detrended waveform, filtering out the mean value of the curvature signal (“DC component”). This operation produced curvature spectra with null power spectral density along the zero-order harmonic (*k* = 0) (Fig. 5a).

### Singular spectrum analysis (SSA)

The SSA is a tenchinque often employed in multivariate time series analysis, aiming at characterizing the signature of regular dynamics underlying on the investigated processes^32^. In the present study we applied SSA to full-meander curvature series^29^ (${\mathscr{C}}_{F}({s}_{n{\prime} }),n{\prime} =\mathrm{0,}\,\mathrm{1,}\ldots ,N{\prime}$). First, we computed the lag-covariance matrix ${{\bf{C}}}_{{\mathscr{C}}_{F}}$ between the values ${\mathscr{C}}_{F}({s}_{n{\prime} })$ and ${\mathscr{C}}_{F}({s}_{n{\prime} }+m)$, where $m=\mathrm{0,}\,\mathrm{1,}\ldots ,M-1$ is a given integer lag whose maximum value depends on the arbitrary window-size *M* chosen to embed the original signal. The size of *M* was determined based on a trade-off between the information extracted from the SSA (large *M*) and the statistical confidence in that information (large *N*′/*M*). Such a procedure is equivalent to embedding the original data series into an *M*-dimensional vector space, thus creating a matrix of embedded data series **Y**, whose rows are constituted by a sequence of *N*′ − *M* + 1 overlapping views of the original data series through a sliding window of size *M*. The lag-covariance matrix ${{\bf{C}}}_{{\mathscr{C}}_{F}}$ can then be computed as the covariance matrix of **Y**. Second, the eigenvalues *λ*_*k*_ and the corresponding eigenvectors *ρ*_*k*_ ($k=\mathrm{1,}\ldots ,M$), of the *M* × *M* lag-covariance matrix ${{\bf{C}}}_{{\mathscr{C}}_{F}}$ were calculated using Singular Value Decomposition (SVD), and sorted in decreasing order based on *λ*_*k*_. Then, the Principal Components (PCs) of the original data series were obtained by projecting the embedded data series **Y** along the eigenvectors *ρ*_*k*_ of the lag-covariance matrix. Finally, the PCs were projected back onto *ρ*_*k*_, thus producing series of Reconstructed Components (RCs) of the pristine signal in the original data domain. A comparison between the *M* Singular Values (SVk = ${\lambda }_{k}^{\mathrm{1/2}}$) of the lag-covariance matrix yielded a reduction of the original data into oscillatory (fundamental) and noisy (higher-order) components, thus revealing the complexity of analyzed series^29,32^.

### Multivariate singular spectrum analysis (M-SSA)

Multivariate Singular Spectrum Analysis is the extension of SSA to a *L*-multivariate input signal. Similarly to SSA, the M-SSA allows one to decompose the original signal into (*L* · *M*) spectral components and to account for cross-correlation in the pristine data. The (*L* · *M*) × (*L* · *M*) lag-covariance matrix is calculated for M-SSA by including both the auto- and cross-covariance function of the original signal. Hence, possible oscillatory components identified in the (*L* · *M*) RCs are common to each of the *L* series constituting the analysed data. Here we considered *L* = 3 morphometric features, namely the half-meander intrinsic wavelength (${\ell }_{H}$), the half-meander asymmetry index (${\mathscr{A}}_{H}$) and the half-meander sinuosity (*σ*_*H*_). M-SSA was employed to analyse series of *M*-adjacent half-meanders^29^, with *M* equal to 2, 5 and 8.

### Principal component analysis (PCA)

The PCA is a statistical technique that transforms high-dimensional datasets of possibly correlated variables into lower-dimensional subspaces, and has already been employed for the analysis of river planforms^28,29,33^. Given a (*p* × *q*) data matrix **X**, where *p* is equal to the number of original samples and *q* represents the number of considered variables, and in order to prevent PCA from giving more emphasis to variables exhibiting higher variance, data in **X** were previously standardized such that all variables have zero mean and unit variance. Consequently, the covariance matrix of **X** corresponds to its correlation matrix **R**. PCA thus transforms the original data **X** into a lower-dimensional subspace as **X** = **S** · **P**′ + **E**, where **P**′ is the projection matrix, the columns of **S**, named score vectors (*s*_*a*_), define the coordinates of the original data in the PC subspace, and **E** is the matrix of residual (i.e., the differences between the original and projected data). The rows of the projection matrix **P**′, named loading vectors **a**, define the Principal Component subspace. The axis directions of the PC subspace correspond to the eigenvectors of the correlation matrix **R**. Being **R** symmetric, the loading vectors **a** are orthogonal by definition and represent the directions along which most of the variance in the original dataset is contained. The loading vectors **a** are usually sorted in descending order based on their corresponding eigenvalues, assuming that **a** associated with the largest eigenvalues contains the most useful information. Several plots are generally employed to visualize and better understand results of PCA. Particularly, the score plot (Fig. 6a) shows the score vectors in the Principal Components subspace. Typically, the first few principal components suffice in reproducing the most dominant pattern in **X**. Conversely, the biplot (Fig. 6a) consists of the combined representation of the principal component loadings and scores, allowing one to visualize which variables are responsible for the separation between different classes in the dataset. In order to fit in the loading space, scores have to be divided by the maximum absolute value of all scores and multiplied by the length of the corresponding loading vectors.

## Supplementary information

Supplementary Information.

## Data Availability

The datasets generated and/or analysed during the current study are freely available at https://github.com/alvitello/Tidal_Fluvial_Meanders.
