# Supplementary material for: Remotely-sensed planform morphologies reveal fluvial and tidal nature of meandering channels
Source: Sci Rep. 2020 Jan 9;10:54. doi: 10.1038/s41598-019-56992-w (PMC6952398; doi:10.1038/s41598-019-56992-w)
Supplement: Supplementary file 1 — Supplementary Information. [file 41598_2019_56992_MOESM1_ESM.pdf]

SUPPLEMENTARY INFORMATION
Remotely-sensed planform morphologies reveal fluvial and tidal nature of meandering channels

Alvise Finotello[1,*], Andrea DÁlpaos[1,], Manuel Bogoni[2], Massimiliano Ghinassi[1], and Stefano Lanzoni[2]**

[1]Department of Geosciences, University of Padova, via G.Gradenigo, 6, Padova, PD I-35131, Italy
[2]Department ICEA, University of Padova, via Loredan, 20, Padova, PD I-35131, Italy

[*]alvise.finotello@unipd.it

[**]andrea.dalpaos@unipd.it

Content

- Table S1
- Figure S1

Table S1: List of tidal and fluvial reaches included in the dataset

Type	Name	Location	Bends	Upstream Point		Downstream Point		Source
				Lat (°)	Long (°)	Lat (°)	Lon (°)	
F	Beaver	Canada	65	54.2759532	-110.085311	54.276738	-109.806571	© 2016 Google, DigitalGlobe
F	Bravo	Mexico/USA	504	26.3917015	-98.9921725	25.952814	-97.1488527	© 2016 Google, DigitalGlobe
F	Chet	Russia	78	56.9212667	87.5104722	56.8807861	87.2661861	© 2016 Google, Getmapping plc
F	Chinchaga	Canada	114	58.5995917	-118.342819	58.8704167	-118.315772	© 2016 Google, Cnes/Spot Image
F	Chulym	Russia	354	56.677609	90.5456672	56.9822823	86.8538009	© 2016 Google, Cnes/Astrium
F	Curucà 1	Brazil	208	-5.89486948	-72.1460794	-5.40288789	-72.1243004	© 2016 Google, Cnes/Spot Image
F	Curucà 2	Brazil	204	-5.24650786	-72.0256226	-4.52597928	-71.4045286	© 2016 Google, Cnes/Spot Image
F	Darling	Australia	479	-32.5280225	142.367171	-34.0846329	141.919923	© 2016 Google, Digital Globe
F	Dulgalakh	Russia	65	67.4265016	132.542146	67.4701683	133.272379	© 2016 Google, Digital Globe
F	Envira	Brazil	153	-8.10899907	-70.3503991	-7.5023306	-70.0632939	© 2016 Google, Digital Globe
F	Javari	Brazil/Perù	194	-4.95751495	-72.5295212	-4.50166231	-71.81738	© 2016 Google, Landsat/Copernicus
F	Juruà 1	Brazil	210	-7.16541952	-71.8216636	-6.79500953	-70.7075243	© 2016 Google, Landsat
F	Juruà 2	Brazil	122	-6.56091028	-69.7530892	-6.46518528	-68.3568182	© 2016 Google, Landsat
F	Juruà 3	Brazil	223	-5.99664528	-67.8375618	-3.62300687	-66.0969369	© 2016 Google, Landsat
F	Kemchug 1	Russia	151	56.8602508	91.8561984	57.1854299	90.702364	© 2016 Google, Digital Globe
F	Kemchug 2	Russia	148	56.1891069	91.6014545	56.858205	91.8602946	© 2016 Google, Digital Globe
F	Kipo Kulary	Russia	242	57.0204656	74.1530056	57.9899007	68.9774677	© 2016 Google, Digital Globe
F	Kwango 1	Angola	98	-9.632625	18.3923472	-9.31084455	18.2605109	© 2016 Google, CNES/Astrium
F	Kwango	Angola	243	-10.0601199	18.700228	-9.632625	18.3923472	© 2016 Google, CNES/Astrium
F	Kyaukgy	Myanmar	166	18.1873444	96.7908556	17.9581722	96.8490361	© 2016 Google, CNES/Astrium
F	Mississippi 1	USA	85	36.5036094	-89.4122858	33.8208077	-91.0485219	© 2016 Google, Landsat/Copernicus
F	Mississippi 2	USA	113	33.8023842	-91.0414646	30.0065178	-90.8304361	© 2016 Google, Landsat/Copernicus

F	Murray 1	Australia	342	-36.0078006	145.974861	-35.8543651	145.004913	© 2016 Google, CNES/Astrium
F	Murray 2	Australia	230	-36.0835025	144.841575	-35.7371846	144.294001	© 2016 Google, CNES/Astrium
F	Murray 3	Australia	104	-34.7287556	143.218156	-34.5837472	142.786858	© 2016 Google, CNES/Astrium
F	Nan	Thailand	111	16.8985028	100.230208	16.5188861	100.325689	© 2016 Google, Digital Globe
F	Okawango	Angola	65	-17.8294026	20.4482761	-17.9739323	20.7655823	© 2016 Google, CNES/Astrium
F	Orthon	Bolivia	443	-10.9957278	-67.4326444	-10.815325	-66.0290889	© 2016 Google, CNES/Spot Image
F	Purus 1	Brazil	182	-7.2976614	-64.8482637	-5.77590503	-64.3862845	© 2016 Google, Landsat/Copernicus
F	Purus 2	Brazil	133	-5.76491423	-64.3898896	-5.07121634	-62.9607449	© 2016 Google, Landsat/Copernicus
F	Tarauacà 1	Brazil	144	-8.05305467	-70.6975173	-7.66969521	-70.5614215	© 2016 Google, Digital Globe
F	Tarauacà 2	Brazil	144	-7.665256	-70.5601418	-7.48830824	-70.0722628	© 2016 Google, Landsat/Copernicus
F	Tarauacà 3	Brazil	114	-7.47326714	-70.0648005	-6.77134609	-69.7646868	© 2016 Google, Digital Globe
F	Tym 1	Russia	274	60.2393387	85.1074019	60.3755083	84.2941291	© 2016 Google, Landsat/Copernicus
F	Tym 2	Russia	178	60.3651267	84.0867962	59.957675	82.3845398	© 2016 Google, Landsat/Copernicus
F	Tym 3	Russia	114	59.9025032	81.8438673	59.5676984	80.4264604	© 2016 Google, Landsat/Copernicus
F	Vakh	Russia	202	61.4204616	82.9134597	61.2241679	80.9942381	© 2016 Google, Landsat/Copernicus
F	Yana	Russia	189	67.4724932	133.283758	68.2141769	134.762418	© 2016 Google, Landsat/Copernicus
T	Mourilyan Harbour	Australia	25	-17.6206815	146.082735	-17.6101106	146.115074	© WorldImagery-DigitaGlobe
T	Dei Lovi Channel 1	Italy	23	45.6472072	12.979739	45.648296	12.9800436	© WorldImagery-Microsoft
T	Dei Lovi Channel 2	Italy	24	45.6449425	12.976502	45.6454153	12.9764952	© WorldImagery-Microsoft
T	Sindacale Channel	Italy	19	45.7107163	12.8646187	45.6169926	12.9123164	© WorldImagery-Microsoft
T	Dongying	China	21	37.7148375	119.146217	37.658617	119.135927	© WorldImagery-CNES/AirbusDS
T	Luoyuan Bay	China	27	26.3663271	119.633496	26.3687986	119.672214	© WorldImagery-CNES/AirbusDS
T	Stang	France	28	47.6238262	-3.02092334	47.6195318	-3.02754369	© WorldImagery-Microsoft
T	Rade de Morlaix	France	25	48.6338097	-3.85026496	48.6328447	-3.85196399	© WorldImagery-Microsoft
T	Str el Istrec	France	27	47.7260728	-3.12702801	47.7173762	-3.10321135	© WorldImagery-Microsoft
T	Etiere de la Barre	France	102	47.4047599	-2.35680907	47.4216054	-2.46453992	© WorldImagery-Microsoft

T	Etiere do Goilè	France	30	47.4104953	-2.45616626	47.4168753	-2.44695944	© WorldImagery-Microsoft
T	Baie du Lindin	France	45	47.5428998	-2.81873712	47.5467598	-2.82598082	© WorldImagery-Microsoft
T	Gulf of Morbihan	France	21	47.5399615	-2.78080596	47.5460218	-2.77412886	© WorldImagery-Microsoft
T	Scorton Creek 1	USA	61	41.7262749	-70.3965255	41.7218944	-70.3452908	© WorldImagery-USDA FSA
T	Scorton Creek 2	USA	50	41.7278053	-70.4208178	41.7483909	-70.4282495	© WorldImagery-USDA FSA
T	Chase Garen Creek	USA	107	41.7330848	-70.2015189	41.7250524	-70.2380801	© WorldImagery-USDA FSA
T	Baie d'Enfer	France	37	48.8368347	-3.20431942	48.8405183	-3.20522683	© WorldImagery-Microsoft
T	Riviere de Sarzeau	France	34	47.5477152	-2.66258776	47.527673	-2.61879919	© WorldImagery-Microsoft
T	Rivier de l'Epinay Surzur	France	31	47.5501712	-2.62892825	47.5295143	-2.61344281	© WorldImagery-Microsoft
T	Ambatomilahy	Madagascar	42	-17.4453343	43.9747288	-17.4016692	43.9767929	© WorldImagery-DigitalGlobe
T	Sungai Santi	Malaysia	30	1.46737493	104.144574	1.42307475	104.146713	© WorldImagery-DigitalGlobe
T	Sungai Kerisek	Malaysia	25	5.62270355	100.374915	5.65519335	100.383908	© WorldImagery-DigitalGlobe
T	Sungai Tinggi	Malaysia	23	4.58177178	100.673939	4.64697548	100.621056	© WorldImagery-DigitalGlobe
T	Htaung Kwin 1	Myanmar	34	15.8970487	95.5660354	15.852572	95.3017873	© WorldImagery-DigitalGlobe
T	Htaung Kwin 2	Myanmar	31	15.9588447	95.5700334	15.868668	95.3014037	© WorldImagery-DigitalGlobe
T	Htaung Kwin 3	Myanmar	25	15.931703	95.0616683	15.7727606	94.9888364	© WorldImagery-DigitalGlobe
T	Santa Luzia 2	Portugal	34	37.093131	-7.6532429	37.0990013	-7.65814119	© WorldImagery-Microsoft
T	Santa Luzia 2	Portugal	20	37.0808619	-7.67801404	37.0872599	-7.68008735	© WorldImagery-Microsoft
T	Manily Kamcatka	Russia	23	62.391663	165.035446	62.4052687	165.052551	© WorldImagery
T	Marismas de Isla Cristina	Spain	23	37.2199843	-7.27658855	37.2058346	-7.32198386	© WorldImagery-IGN/CNIG
T	Marismas de Isla Cristina 2	Spain	30	37.2090707	-7.30593461	37.2046721	-7.31182559	© WorldImagery-IGN/CNIG
T	Cadiz Bay	Spain	26	36.5314543	-6.22718051	36.5273809	-6.22664205	© WorldImagery-IGN/CNIG
T	Rio de la Bota	Spain	21	37.2136439	-7.02313109	37.2073251	-6.97787744	© WorldImagery-IGN/CNIG
T	Hai Lam	Thailand	28	13.2739717	99.857132	13.2653862	99.9418714	© WorldImagery-DigitalGlobe
T	Tiang	Thailand	20	6.67748	99.9410091	6.66962321	99.9272133	© WorldImagery-DigitalGlobe
T	Hat Sai Ri	Thailand	25	10.3936728	99.2147404	10.3669242	99.2147956	© WorldImagery-DigitalGlobe

T	Tha Taphao	Thailand	28	12.1957674	102.494367	12.1614123	102.473084	© WorldImagery-DigitalGlobe
T	Nam Chiao	Thailand	22	12.1803908	102.452518	12.1646261	102.465271	© WorldImagery-DigitalGlobe
T	Monie Creek	USA	31	38.22521	-75.752248	38.2306495	-75.822344	© WolrdImagery-USDA FSA
T	Tuckahoe	USA	45	39.3065659	-74.8180276	39.2885488	-74.6528424	© WolrdImagery-USDA FSA
T	Lakes Creek	USA	45	39.3435766	-74.6415487	39.3254583	-74.6635727	© WolrdImagery-USDA FSA
T	China Camp 1	USA	39	38.0080337	-122.488975	38.0100995	-122.484363	© WorldImagery-SFEI -Quantum Spatial
T	China Camp 2	USA	32	38.0115437	-122.498182	38.0154738	-122.497214	© WorldImagery-SFEI -Quantum Spatial
T	Pagliaga 1	Italy	21	45.510739	12.374582	45.5064434	12.3738702	© WorldImagery-Microsoft
T	Pagliaga 2	Italy	24	45.5192427	12.3696871	45.512902	12.3713845	© WorldImagery-Microsoft
T	Tessera 1	Italy	29	45.4894881	12.3193763	45.4828551	12.3209192	© WorldImagery-Microsoft
T	Tessera 2	Italy	26	45.4856439	12.312369	45.4843341	12.3168496	© WorldImagery-Microsoft
T	Tessera 3	Italy	32	45.48481	12.312375	45.4856952	12.3154766	© WorldImagery-Microsoft
T	Tessera 4	Italy	31	45.4853688	12.3125456	45.4829652	12.3123595	© WorldImagery-Microsoft
T	Tessera 5	Italy	26	45.4808528	12.3089558	45.4804666	12.309012	© WorldImagery-Microsoft
T	Lazzareto Nuovo	Italy	26	45.4589725	12.3845916	45.457623	12.3858777	© WorldImagery-Microsoft
T	Song Dong Tranh	Vietnam	21	10.5506858	106.791718	10.4097749	106.862543	© WorldImagery-DigitalGlobe
T	Thi Vai	Vietnam	23	10.6285206	106.903069	10.5253527	107.00998	© WorldImagery-DigitalGlobe
T	Mellum	Germany	35	53.7224173	8.14689401	53.7225604	8.1615012	© WorldImagery-DigitalGlobe
T	Nordeney	Germany	30	53.7181549	7.30527091	53.7121732	7.315367	© WorldImagery-DigitalGlobe
T	Skallingen 1	Denmark	36	55.4950211	8.29139214	55.4946754	8.30631926	© WorldImagery-DenmarkImagery
T	Skallingen 2	Denmark	31	55.5052757	8.2735352	55.5095892	8.2837988	© WorldImagery-DenmarkImagery
T	Langeoog	Germany	51	53.7277485	7.47353972	53.7259237	7.48267214	© WorldImagery-USDA FSA

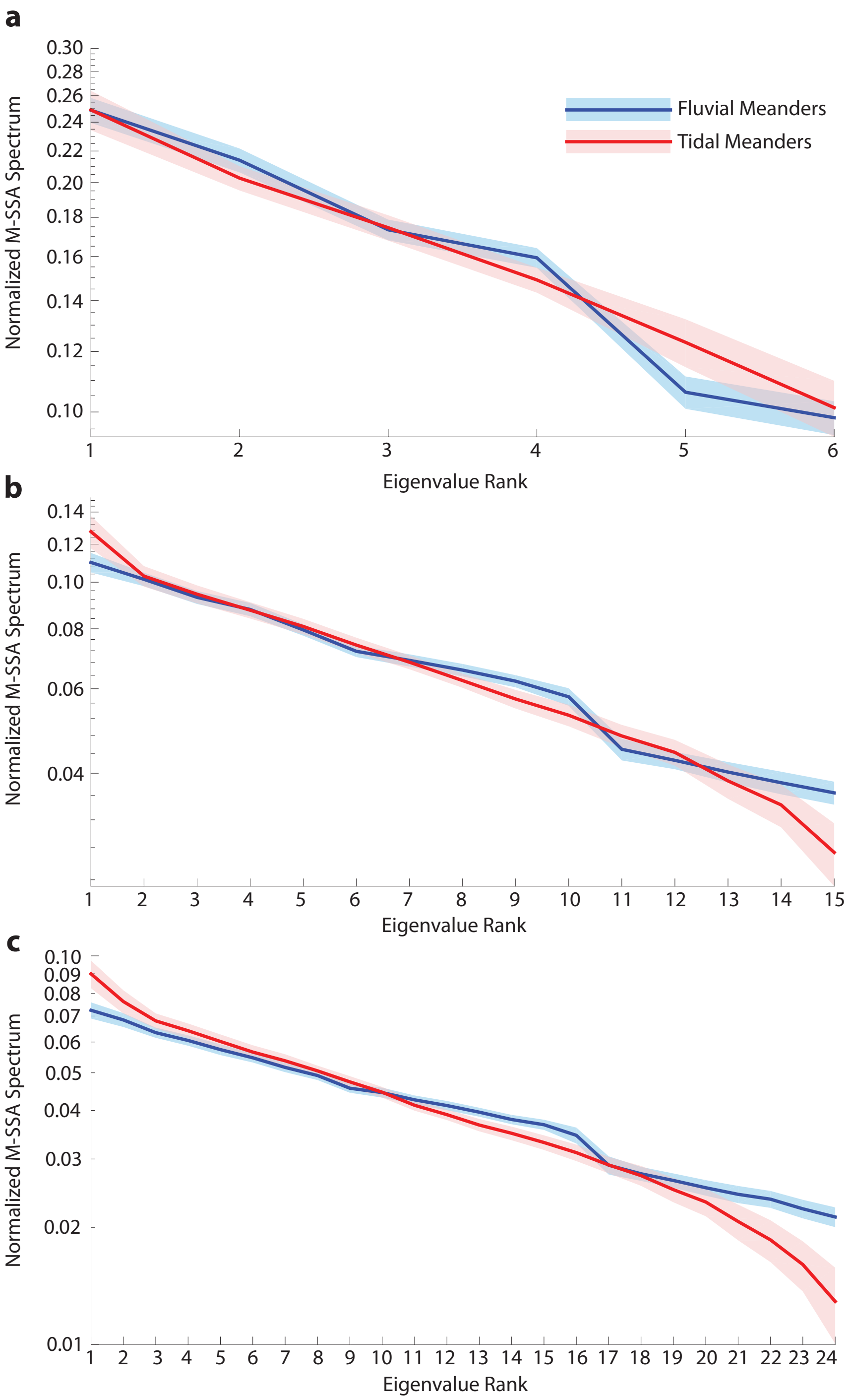

Figure S1: M-SSA eigenvalue spectra of tidal and fluvial meanders. (a) M-SSA spectra obtained considering 2 consecutive half meanders, (b) M-SSA spectra obtained considering 5 consecutive half meanders, (c) M-SSA spectra obtained considering 8 consecutive half meanders. The units of abscissa are M-SSA component number (eigenvalue rank), while the ordinate show the variance contributed by each M-SSA component. Solid lines represent averaged values. The displayed intervals correspond to one standard deviation.
